# Work disability remains a major problem in rheumatoid arthritis in the 2000s: data from 32 countries in the QUEST-RA Study

**DOI:** 10.1186/ar2951

**Published:** 2010-03-12

**Authors:** Tuulikki Sokka, Hannu Kautiainen, Theodore Pincus, Suzanne MM Verstappen, Amita Aggarwal, Rieke Alten, Daina Andersone, Humeira Badsha, Eva Baecklund, Miguel Belmonte, Jürgen Craig-Müller, Licia Maria Henrique da Mota, Alexander Dimic, Nihal A Fathi, Gianfranco Ferraccioli, Wataru Fukuda, Pál Géher, Feride Gogus, Najia Hajjaj-Hassouni, Hisham Hamoud, Glenn Haugeberg, Dan Henrohn, Kim Horslev-Petersen, Ruxandra Ionescu, Dmitry Karateew, Reet Kuuse, Ieda Maria Magalhaes Laurindo, Juris Lazovskis, Reijo Luukkainen, Ayman Mofti, Eithne Murphy, Ayako Nakajima, Omondi Oyoo, Sapan C Pandya, Christof Pohl, Denisa Predeteanu, Mjellma Rexhepi, Sylejman Rexhepi, Banwari Sharma, Eisuke Shono, Jean Sibilia, Stanislaw Sierakowski, Fotini N Skopouli, Sigita Stropuviene, Sergio Toloza, Ivo Valter, Anthony Woolf, Hisashi Yamanaka

**Affiliations:** 1 Jyväskylä Central Hospital, Keskussairaalantie 19, 40620 Jyväskylä, Finland; 2 Medcare Oy, Hämeentie 1, 44100 Äänekoski, Finland; 3 New York University Hospital for Joint Diseases, 301 East 17 Street, New York, NY 10003, USA; 4 Department of Rheumatology and Clinical Immunology F02.127, University Medical Center Utrecht, P.O. Box 85500, 3508 GA Utrecht, The Netherlands; 5 Department of Immunology, Sanjay Gandhi Postgraduate Institute of Medical Sciences, Lucknow, 226014, India; 6 Department of Internal Medicine II, Rheumatology, Schlosspark-Klinik Teaching Hospital of the Charité, University Medicine Berlin, Heubnerweg 2, 14059 Berlin, Germany; 7 Medical Faculty of Latvia University, P. Stradina Clinical University Hospital, Pilsonu Street 13, LV 1002, Riga, Latvia; 8 Rheumatology Department, Dubai Bone and Joint Center, Al Razi Building, DHCC, PO Box 118855, Dubai 118855, United Arab Emirates; 9 Uppsala University Hospital, Department of Medical Sciences, Uppsala University, 751 85 Uppsala, Sweden; 10 Sección de Reumatologia, Hospital General de Castellón, Avda Benicasim s/n, 12004 - Castellon, Spain; 11 CentraCare, 1200 6th Avenue North, St. Cloud, MN 56301, USA; 12 Serviço de Reumatologia - Hospital Universitário de Brasília, SGAN 605, Av. L2 Norte Brasília, Brazil; 13 Rheumatology Institut, Srpskih Junaka 2, 18205 Niška Banja, Serbia; 14 Rheumatology & Rehabilitation, Assiut University Hospital, Assiut University, Assiut 71111, Egypt; 15 School of Medicine, Catholic University of the Sacred Heart, Via Moscati 31, 00168 Rome, Italy; 16 Department of Rheumatology, Kyoto First Red Cross Hospital, 15-749, Mon-machi, Higashiyama-ku, Kyoto, Japan; 17 Department of Rheumatology, Semmelweis University, H-1025 Budapest Árpád f.u.7., Hungary; 18 Department of Physical Medicine and Rehabilitation, Division of Rheumatology, Gazi University, 06530 Ankara, Turkey; 19 Faculté de Médecine et de Pharmacie, Route de la Plage, Rabat, Morocco; 20 Al-Azhar University, 14 Mustafa Darwish Street, Nasr City, Cairo, Egypt; 21 Department of Rheumatology, Service box 416, N-4604 Kristiansand. S, Norway; 22 King Christian the Xth Hospital, Toldbodgade 3, 6300 Gråsten, Denmark; 23 Clinica de Medicina Interna si Reumatologie, Spitalul Clinic Sf Maria, B-dul Ion Mihalache 37-39 Sector 4, Bucuresti, Romania; 24 Department of Early Arthritis, Institute of Rheumatology, Kashirskoye shosse, 34a, Moscow, 115522, Russia; 25 Tartu University Hospital, Puusepa str. 6, Tartu 50408 Estonia; 26 Faculdade de Medicina da Universidade de São Paulo-FMUSP, Av Dr Arnaldo 455, CEP01246-903, São Paulo, Brazil; 27 Rheumatology Section, Riverside Professional Center, 31 Riverside Drive, Sydney, NS, B1S 3N1, Canada; 28 Satakunta Central Hospital, Rauman aluesairaala, Steniuksenkatu 2, 26100 Rauma, Finland; 29 American Hospital Dubai, P.O. Box 5566, Dubai, United Arab Emirates; 30 Connolly Hospital, Waterville Road, Blanchardstown, Dublin 15, Ireland; 31 Institute of Rheumatology, Tokyo Women's Medical University, 10-22 Kawada-cho, Shinjuku-ku, Tokyo, Japan; 32 Kenyatta National Hospital, Hospital Road,, PO Box 19701-00202, Nairobi, Kenya; 33 Rheumatic Disease Clinic, 4th floor, Vedanta Institute of Medical Sciences Navrangpura, Ahmedabad 380009, Gujarat, India; 34 Rheumatology Department, University Clinical Center of Kosova, Kodra e diellit, Rr. II, Lamela 11/9, Prishtina, 10 000, Kosova; 35 Department of Clinical Immunology, Jaipur Hospital, Lal Kothi, Jaipur Pin-302021, India; 36 Shono Rheumatism Clinic, 1-10-27 Nishi-shin, Sawara-ku, Fukuoka, Japan; 37 Service de Rhumatologie, CHU de Strasbourg, Hôpital Hautepierre, Avenue Molière, BP 49, 67098 Strasbourg, France; 38 Department of Rheumatology and Internal Diseases, Medical University in Bialystok, 24a Maria Sklodowska-Curie Street, 15-276 Bialystok, Poland; 39 Harokopio University and Euroclinic of Athens, Athanasiadou 7-9, 11521 Athens, Greece; 40 Institute of Experimental and Clinical Medicine at Vilnius University, 3 Universiteto St, LT-01513 Vilnius, Lithuania; 41 Division of Rheumatology, Hospital San Juan Bautista, Avenida Illia 200, Catamarca, CP 4700, Argentina; 42 Center for Clinical and Basic Research, Tallinn, Pärna 4, 10128 Tallinn, Estonia; 43 Duke of Cornwall Rheumatology Unit, Royal Cornwall Hospital, Truro, Cornwall, TR1 3LJ, UK

## Abstract

**Introduction:**

Work disability is a major consequence of rheumatoid arthritis (RA), associated not only with traditional disease activity variables, but also more significantly with demographic, functional, occupational, and societal variables. Recent reports suggest that the use of biologic agents offers potential for reduced work disability rates, but the conclusions are based on surrogate disease activity measures derived from studies primarily from Western countries.

**Methods:**

The Quantitative Standard Monitoring of Patients with RA (QUEST-RA) multinational database of 8,039 patients in 86 sites in 32 countries, 16 with high gross domestic product (GDP) (>24K US dollars (USD) per capita) and 16 low-GDP countries (<11K USD), was analyzed for work and disability status at onset and over the course of RA and clinical status of patients who continued working or had stopped working in high-GDP versus low-GDP countries according to all RA Core Data Set measures. Associations of work disability status with RA Core Data Set variables and indices were analyzed using descriptive statistics and regression analyses.

**Results:**

At the time of first symptoms, 86% of men (range 57%-100% among countries) and 64% (19%-87%) of women <65 years were working. More than one third (37%) of these patients reported subsequent work disability because of RA. Among 1,756 patients whose symptoms had begun during the 2000s, the probabilities of continuing to work were 80% (95% confidence interval (CI) 78%-82%) at 2 years and 68% (95% CI 65%-71%) at 5 years, with similar patterns in high-GDP and low-GDP countries. Patients who continued working versus stopped working had significantly better clinical status for all clinical status measures and patient self-report scores, with similar patterns in high-GDP and low-GDP countries. However, patients who had stopped working in high-GDP countries had better clinical status than patients who continued working in low-GDP countries. The most significant identifier of work disability in all subgroups was Health Assessment Questionnaire (HAQ) functional disability score.

**Conclusions:**

Work disability rates remain high among people with RA during this millennium. In low-GDP countries, people remain working with high levels of disability and disease activity. Cultural and economic differences between societies affect work disability as an outcome measure for RA.

## Introduction

Work disability is a major consequence of rheumatoid arthritis (RA) [1-4]. Although RA is cumulative over time, 20% to 30% of patients become permanently work-disabled in the first 2 to 3 years of the disease [5]. Rapid remission in early disease appears to be a beneficial strategy against work disability in RA [6].

The availability of biologic agents during the past decade has led to expectations of reduced work disability rates in RA [7], according to observations in clinical trials [8-12]. However, reports of clinical cohorts indicate that work disability remains a major problem in RA [13-16]. Possible explanations are that the timing of biologic agents after joint damage is seen may be too late in many cases or that the use of biologic agents is unusual in many countries for financial reasons or both [17].

The risk of work disability in RA is associated not only with traditional articular, radiographic, and laboratory measures of disease activity and severity but as much or more with demographic, socioeconomic, vocational, functional, and social policy variables [1,18]. Although work disability is one of the most important outcomes in RA, cultural and economical differences between societies [19] may compromise its value as an outcome measure.

Most studies concerning work disability in RA have been conducted in North America and Western Europe, and little is known about the employability of RA patients in other countries. A multinational database, Quantitative Standard Monitoring of Patients with Rheumatoid Arthritis (QUEST-RA) [20,21], was established to assess 100 unselected consecutive RA patients per clinic in different countries, including countries outside Western Europe and North America. In June 2009, the QUEST-RA database included 8,039 patients from 86 clinics in 32 countries. The QUEST-RA database was analyzed for work status at the onset of RA and discontinuation of work due to RA, as recalled by the patients. Current clinical status of patients who continued to work or who had stopped working in high-gross domestic product (high-GDP) and low-GDP countries was analyzed.

## Materials and methods

### Establishment of database

QUEST-RA was established in 2005 with the objectives to promote quantitative assessment in usual rheumatology care and to develop a baseline cross-sectional database of consecutive RA patients seen outside of clinical trials in regular care in many countries. Three or more rheumatologists were asked to enroll 100 consecutive unselected patients in each country [20]. As of June 2009, the program enrolled 8,039 patients from 86 sites in 32 countries, including Argentina, Brazil, Canada, Denmark, Egypt, Estonia, Finland, France, Germany, Greece, Hungary, India, Ireland, Italy, Japan, Kenya, Kosovo, Latvia, Lithuania, Morocco, The Netherlands, Norway, Poland, Romania, Russia, Serbia, Spain, Sweden, Turkey, United Arab Emirates, the UK, and the US [21].

### Ethics committee approvals

The study was carried out in compliance with the Declaration of Helsinki. Ethics committees or internal review boards of participating institutes approved the study, and written informed consent was obtained from the patients.

### Physician assessment measures

All patients were assessed according to a standard protocol to evaluate RA (SPERA) [22]. The physician review included four RA Core Data Set measures: a formal examination of swollen joints (swollen joint count using 28 joints, or SJC28) and tender joints (tender joint count using 28 joints, or TJC28), global estimate of disease activity, and erythrocyte sedimentation rate (ESR). Rheumatoid factor (RF) positivity according to local reference values was reported as well as whether the patient had radiographic erosions. All courses of disease-modifying antirheumatic drugs (DMARDs) and biologic agents were listed. The number of DMARDs over disease course was calculated.

### Patient self-report questionnaire measures

Patients completed a 4-page self-report questionnaire, which included three RA Core Data Set measures - physical function, pain, and patient global estimate (PTGL) - as well as demographic data, fatigue, and psychological distress. A standard Health Assessment Questionnaire (HAQ) [23] assesses physical function in activities of daily living and has four response categories: 0 = without any difficulty, 1 = with some difficulty, 2 = with much difficulty, 3 = unable to do. Visual analog scales (VASs) (0 = best to 10 = worst) were completed for pain, fatigue, and PTGL. Psychological distress was queried as the capacity to cope with sleep, stress, anxiety, and depression in the HAQ format with response options 0 to 3 (seen above) for each question. The mean of the responses of these four questions was calculated for PS-HAQ (Psychological HAQ) of 0 to 3 [24].

Data concerning work or disability status were based on a patient self-report questionnaire that included queries about work status at the time of the first symptoms of RA and currently and that had the following response alternatives: working full-time, working part-time, unemployed, student, homemaker, retired, and disabled. Full-time workers, part-time workers, students, and unemployed individuals were classified as working in these analyses as these groups had the potential to be employed in the workforce. Homemakers, retirees, and disabled individuals were classified as non-working, although individuals in these groups may perform valuable non-paid work. Patient self-perceived work disability was queried with the questions, 'Are you work-disabled because of RA?' (with response options 'yes' or 'no') and 'If so, since when?'

### Gross domestic product

The GDP of each country in 2005 was obtained from a database of the International Monetary Fund [25] and is expressed in units of 1,000 US dollars (USD) per capita. GDP ranged from 3.5 to 49K USD but was either less than 11K USD or greater than 24K USD in all countries. In this report, the 16 countries with a GDP per capita of greater than 24K USD are classified as 'high-GDP' whereas the 16 countries with a GDP per capita of less than 11K USD are classified as 'low-GDP'.

### Statistical methods

Descriptive results are presented as mean, median, and percentages. The disease activity score using 28 joint counts (DAS28) [26] was calculated from the formula 0.56*v(TJC28) + 0.28*v(SJC28) + 0.70*ln(ESR) + 0.014*(PTGL) and ranged from 0 to 9.1 (low to high). Comparisons of demographic variables, clinical characteristics, RA disease activity measures, and treatment-related variables were performed using parametric and non-parametric tests for continuous variables and the chi-square test for categorical variables. Kaplan-Meier statistics were applied; those who reached age 65 after the onset of RA were censored at that date.

Regressions were performed to analyze which demographic and clinical measures were independently significant to identify work disability status. Variables in the model included age, sex, education level, HAQ physical function, radiographic erosions, RF, ESR, and SJC28. Data of all individuals from all countries were pooled together; regressions were performed in four independent categories for all countries: in low-GDP countries, with onset prior to 2000 and after 2000, and in high-GDP countries, with onset prior to 2000 and after 2000. The year 2000 was chosen as a milestone because the first biologic agent for treatment of RA was approved in 1999.

## Results

### Patients

The mean age of 8,039 patients in the QUEST-RA database, from 86 clinics in 32 countries, was 55 years; 80% were females, and the mean disease duration was 11 years (Table 1). Patients in low-GDP countries were younger and were more likely to be female and have RF and radiographic erosions compared with those in high-GDP countries, as described in detail previously [27]. Patients in low-GDP countries had statistically significantly higher disease activity levels of all individual RA Core Data Set measures, including physician-derived measures and patient self-report scores, compared with patients in high-GDP countries. The number of DMARDs taken, and especially the percentage of patients who had ever taken biologic agents, differed significantly, with 31% in high-GDP countries and 9.4% in low-GDP countries (Table 1).

**Table 1:** Patient and disease characteristics in 8,039 patients in the QUEST-RA study in countries with a high (>24K USD) or low (<11K USD) GDP

	All patients			
	
		Countries		
			
	Total	High-GDP	Low-GDP	*P *value^a^
Number	8,039	4,235	3,804	
Patient characteristics				
Female, percentage	80%	76%	85%	< 0.001
Age in years, mean	55	58	52	< 0.001
Disease duration in years, mean	11	11	11	NS
Rheumatoid factor-positive, percentage	75%	73%	77%	< 0.001
Erosive disease, percentage	63%	59%	67%	< 0.001
Disease activity measures				
DAS28 (0 to 9.1), mean	4.4	3.7	5.2	< 0.001
ESR, median	25	18	33	< 0.001
SJC (0 to 28), median	2	1	4	< 0.001
TJC (0 to 28), median	4	2	9	< 0.001
Physician global (0 to 10), median	2.5	1.7	3.9	< 0.001
Patient self-report				
HAQ physical function (0 to 3), median	0.88	0.75	1.3	< 0.001
Pain VAS (0 to 10), median	4.1	3.2	4.7	< 0.001
Patient global VAS (0 to 10), median	4.2	3.2	4.8	< 0.001
Fatigue VAS (0 to 10), median	4.5	3.8	5.0	< 0.001
Psychological HAQ (0 to 3), mean	0.84	0.73	0.99	< 0.001
Treatment-related variables				
Number of DMARDs ever taken, mean	2.7	2.8	2.5	< 0.001
Biologics ever, percentage of patients	23%	31%	9.4%	< 0.001

^a^Unadjusted *P *values are presented. DAS28, disease activity score using 28 joint counts; DMARD, disease-modifying antirheumatic drug; ESR, erythrocyte sedimentation rate; GDP, gross domestic product; HAQ, Health Assessment Questionnaire; NS, not significant; QUEST-RA, Quantitative Standard Monitoring of Patients with Rheumatoid Arthritis; SJC, swollen joint count; TJC, tender joint count; USD, US dollars; VAS, visual analog scale.

### Work status at baseline

At the time of first symptoms, 68% of patients who were 65 years old or younger, including 85% of men (57% to 100% among countries) and 64% of women (19% to 83%), reported that they were working (Table 2). Among women, the proportion working at onset ranged from greater than 80% in Sweden, Finland, Estonia, and Lithuania to not more than 50% in Turkey, Kosovo, Morocco, Greece, and Egypt (Table 2).

**Table 2:** Percentage of patients who were younger than 65 years old and working at the onset of symptoms in the QUEST-RA study in 32 countries

	Percentage working^a ^at onset		
	
Country	Women	Men	All
Sweden	88%	90%	88%
Finland	83%	86%	84%
Estonia	83%	82%	83%
Lithuania	82%	85%	83%
UK	80%	90%	82%
Latvia	79%	100%	83%
USA	79%	93%	83%
France	78%	86%	80%
Denmark	76%	93%	80%
Hungary	75%	73%	75%
Brazil	73%	83%	73%
Germany	72%	89%	75%
Canada	65%	89%	70%
Argentina	64%	100%	67%
Russia	63%	96%	69%
India	62%	93%	67%
Poland	61%	78%	63%
Japan	60%	85%	64%
The Netherlands	60%	82%	67%
Ireland	59%	91%	71%
UAE	59%	100%	64%
Spain	57%	85%	65%
Serbia	56%	70%	57%
Italy	54%	82%	60%
Egypt	50%	73%	51%
Greece	49%	73%	55%
Morocco	42%	100%	48%
Kosovo	23%	57%	28%
Turkey	19%	66%	25%
Norway	NA	NA	NA
Total	64%	85%	68%

NA, not applicable; QUEST-RA, Quantitative Standard Monitoring of Patients with Rheumatoid Arthritis; UAE, United Arab Emirates. ^a^Defined in Materials and methods.

### Rate of work disability

**Figure 1:**
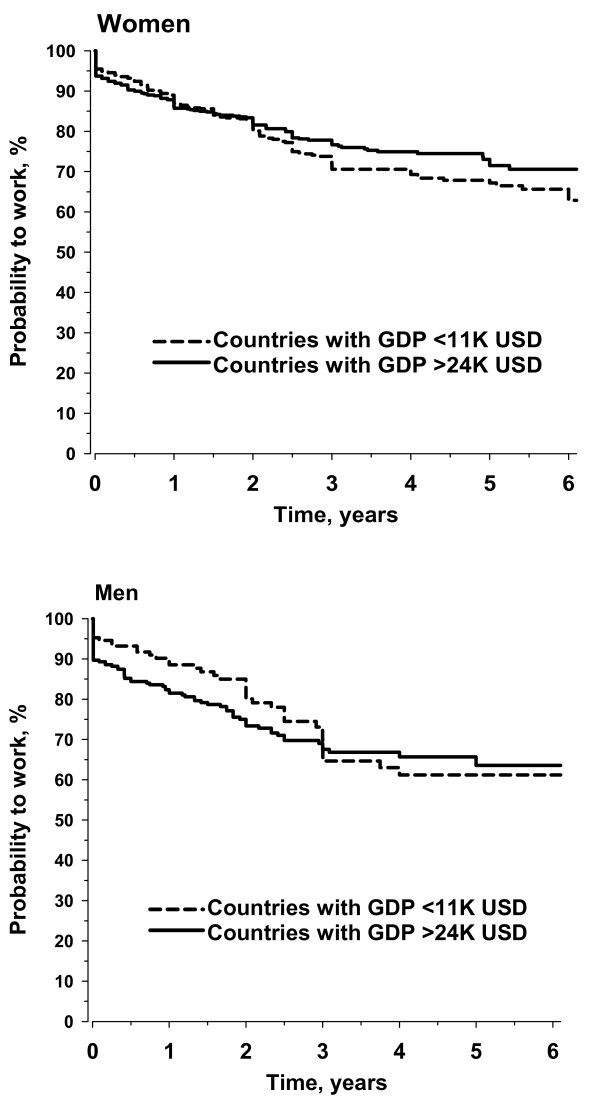
**Kaplan-Meier probability to continue work in women and men who were diagnosed with rheumatoid arthritis in the 2000s in countries with a high or low gross domestic product (GDP)**. USD, US dollars.

A third of the patients (37%) who were younger than 65 years old and working at the onset of symptoms reported that they subsequently became work-disabled because of RA, over the median (interquartile range) observation period of 9 (4 to 16) years. Among 1,754 patients whose symptoms had begun during the 2000s and were working at the baseline, the rates of probability (95% confidence interval) to continue working were 80% (78% to 82%) at 2 years and 68% (65% to 71%) at 5 years, in a similar pattern to women and men in high-GDP and low-GDP countries (Figure 1).

### Clinical status variables in people younger than 65 years old who were working versus those who had stopped working

**Figure 2:**
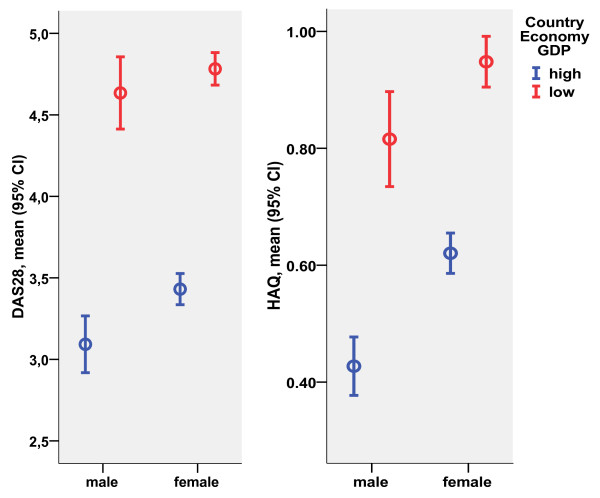
**Disease activity (DAS28) and physical function (HAQ) in men and women who were younger than 65 years old and continued working in high-GDP and low-GDP countries**. CI, confidence interval; DAS28, disease activity score using 28 joint counts; GDP, gross domestic product; HAQ, Health Assessment Questionnaire.

People who had stopped working had poorer clinical status according to all disease activity and patient self-report measures compared with people who were working (Table 3). Although better clinical status was seen in patients who were working than in those who were not working within high-GDP countries and within low-GDP countries, patients who were working in low-GDP countries had significantly better clinical status according to most measures than patients in high-GDP countries who had stopped working (Table 3). Among patients who were younger than 65 years old at the evaluation visit and reported that they are working, the current mean HAQ levels in high-GDP versus low-GDP countries were 0.43 versus 0.82 in men and 0.62 versus 0.95 in women. The mean DAS28 values in high-GDP versus low-GDP countries were, respectively, 3.1 versus 4.6 in men and 3.4 versus 4.8 in women (Figure 2), indicating that women continue working at higher disability and disease activity levels compared with men.

**Table 3:** Patient and disease characteristics in 5,493 individuals younger than 65 years who are working or not working in the QUEST-RA study in countries with a high (>24K USD) or low (<11K USD) GDP

	Individuals younger than 65 years old								
	
	All countries			High-GDP countries			Low-GDP countries		
			
	Working	Not working	*P *value^a^	Working	Not working	*P *value^a^	Working	Not working	*P *value^a^
Number	2,590	2,903		1,436	1,347		1,154	1,556	
Patient characteristics									
Females, percentage	76%	85%	< 0.001	72%	81%	< 0.001	82%	89%	< 0.001
Age in years, mean	46	52	< 0.001	47	54	< 0.001	45	50	< 0.001
Disease duration in years, mean	8.3	11	< 0.001	8.7	12	< 0.001	7.8	11	< 0.001
Rheumatoid factor-positive, percentage	72%	75%	0.004	70%	75%	0.001	75%	76%	0.64
Erosive disease, percentage	53%	65%	< 0.001	51%	62%	< 0.001	56%	69%	< 0.001
Disease activity measures									
DAS28 (0 to 9.1), mean	4.0	4.6	< 0.001	3.3	3.9	< 0.001	4.8	5.2	< 0.001
ESR, median	20	26	< 0.001	14	18	< 0.001	28	32	< 0.001
SJC (0 to 28), median	2	3	< 0.001	1	2	< 0.001	3	4	0.002
TJC (0 to 28), median	3	5	< 0.001	1	2	< 0.001	7	8	< 0.001
Physician global (0 to 10), median	2.1	2.8	< 0.001	1.5	1.9	< 0.001	3.0	3.8	< 0.001
Patient self-report									
HAQ physical function (0 to 3), median	0.63	1.1	< 0.001	0.50	0.88	< 0.001	0.88	1.3	< 0.001
Pain VAS (0 to 10), median	3.2	4.5	< 0.001	2.3	3.8	< 0.001	4.2	4.9	< 0.001
Patient global VAS (0 to 10), median	3.3	4.6	< 0.001	2.4	3.6	< 0.001	4.3	5.0	< 0.001
Fatigue VAS (0 to 10), median	3.9	4.8	< 0.001	3.2	4.2	< 0.001	4.5	5.2	< 0.001
Psychological HAQ (0 to 3), mean	0.79	0.94	< 0.001	0.60	0.82	< 0.001	0.87	1.1	< 0.001
Treatment-related variables									
Number of DMARDs ever taken, mean	2.42	2.73	< 0.001	2.6	3.1	< 0.001	2.2	2.4	< 0.001
Biologics ever, percentage of patients	22%	21%	0.60	32%	35%	0.12	8.5%	9.0%	0.65

^a^Unadjusted *P *values are presented. DAS28, disease activity score using 28 joint counts; DMARD, disease-modifying antirheumatic drug; ESR, erythrocyte sedimentation rate; GDP, gross domestic product; HAQ, Health Assessment Questionnaire; QUEST-RA, Quantitative Standard Monitoring of Patients with Rheumatoid Arthritis; SJC, swollen joint count; TJC, tender joint count; USD, US dollars; VAS, visual analog scale.

### Differences in drug treatments

Among individuals who were younger than 65 years old at the evaluation, the rates of use of biologic agents were higher in those who had discontinued versus continued working and were 39% versus 32% in high-GDP countries and 13% versus 8.3% in low-GDP countries.

### Identifiers of work disability

A series of logistic regressions was performed to analyze the independent capacity of age, sex, education, RF, radiographic erosions, HAQ physical function, ESR, and SJC28 to identify people who reported that they were work-disabled due to RA (Table 4). In the entire database, HAQ physical function, radiographic erosions, and sex were the three significant variables in these regressions (Table 4).

**Table 4:** Regression analyses to identify work disability status in 5,493 individuals younger than 65 years in the QUEST-RA study in 32 countries with a high (>24K USD) or low (<11K USD) GDP

Type of country	Onset of RA	Sex, male	Age	Education lowest 2/3	RF-positive, ever	Erosion-positive, ever	HAQ	ESR	SJC28	Constant
All countries		1.46 (1.26-1.68)	0.99 (0.99-1.0)	1.36 (1.21-1.54)	1.10 (0.96-1.26)	1.74 (1.53-1.96)	2.76 (2.53-3.01)	1.00 (0.99-1.00)	0.99 (0.98-1.00)	0.13
Low-GDP	Pre-2000	2.04 (1.38-3.02)	0.98 (0.97-0.99)	1.22 (0.95-1.58)	1.52 (1.12-2.08)	1.97 (1.40-2.76)	2.65 (2.19-3.19)	1.00 (0.99-1.00)	0.98 (0.96-1.01)	0.32
	Post-2000	1.26 (0.92-1.74)	1.00 (0.99-1.01)	1.65 (1.27-2.14)	1.07 (0.82-1.42)	2.10 (1.64-2.69)	2.33 (1.93-2.81)	1.00 (0.99-1.01)	0.98 (0.95-1.00)	0.07
High-GDP	Pre-2000	1.41 (1.10-1.79)	0.99 (0.98-1.00)	1.40 (1.13-1.73)	0.89 (0.70-1.14)	1.12 (0.89-1.42)	2.94 (2.52-3.43)	1.00 (0.99-1.01)	1.00 (0.98-1.02)	0.25
	Post-2000	1.71 (1.29-2.26)	0.99 (0.98-1.00)	1.43 (1.09-1.87)	0.99 (0.75-1.30)	1.16 (0.90-1.50)	2.89 (2.36-3.54)	1.00 (0.99-1.01)	1.02 (0.99-1.05)	0.15

Odds ratios (95% confidence intervals) are presented. ESR, erythrocyte sedimentation rate; GDP, gross domestic product; HAQ, Health Assessment Questionnaire; QUEST-RA, Quantitative Standard Monitoring of Patients with Rheumatoid Arthritis; RA, rheumatoid arthritis; RF, rheumatoid factor; SJC28, swollen joint count using 28 joints; USD, US dollars.

## Discussion

The multinational QUEST-RA study provides several important observations concerning work-related outcomes in RA: (a) work disability rates remain high among patients with RA during this millennium; (b) major differences are seen in the proportion of women and men working at the onset of RA in different countries; (c) people continue working in low-GDP countries with levels of disease activity as high as or higher than those of work-disabled people with RA in high-GDP countries; and (d) in both high-GDP and low-GDP nations, the most significant identifier of work disability is the HAQ functional disability score.

### Work disability rates remain high among patients with rheumatoid arthritis during this millennium

The availability of biologic agents over the past decade has increased expectations of reduced work disability rates in RA [7], based on results of clinical studies [8-11]. However, reports based on clinical cohorts have not indicated major effects of biologic agents on patients' work status [13-15]. The timing of treatment with biologic agents may not be optimal in many situations, and biologic agents are used infrequently in many countries for financial reasons (Tables 1 and 3) [17]. In QUEST-RA, a greater proportion of those who had discontinued versus continued working had taken biologic agents. Perhaps the early use of biologic agents will be associated with prevention of work disability in the future. However, at this time in actual clinics, the use of biologic agents appears to be primarily a surrogate for disease severity. In an ideal world, a reduction of work disability rates in RA patients might be achieved by initiating early and aggressive treatment within some weeks or months of the first symptoms [28]. The Finnish Rheumatoid Arthritis Combination Trial (FIN-RACo) [6] documented that early remission is critical concerning subsequent work or disability status: All patients who were in remission 6 months after baseline continued to work at 5 years compared with less than 80% with ACR20 (American College of Rheumatology 20% improvement criteria) and ACR50 responses and less than 50% with less than ACR20 responses [6]. FIN-RACo also indicated that traditional DMARDs, without biologic agents, can prevent work disability in early RA.

### Work disability as an outcome measure of rheumatoid arthritis

It has become a fashion to use work disability-related outcome measures in the trials of biologic agents. Reports from clinical trials of biologic agents appear to be based on a traditional 'biomedical model' [29] view that work disability is a direct consequence of severe disease activity and damage. Previous studies document that mean levels of clinical variables in RA patients who were work-disabled in Finland indicated better clinical status than RA patients who were working in the US, and this difference was explained by different social policies regarding work disability [19]. In QUEST-RA, disease activity and disability levels were as high in working people in low-GDP countries as in work-disabled people in high-GDP countries. Thus, the QUEST-RA data extend evidence that macroeconomic variables influence an individual's work status to a greater degree than an individual's disease activity. The majority of patients with RA are women, a low proportion of whom are employed in certain countries at disease onset. In the present study, women continued working with higher scores of disease activity and functional disability compared with men (Figure 2). Cultural and socioeconomic differences between societies affect work disability as an outcome measure. In the RA population, losses in household productivity may be a more important matter than disability at paid work; household productivity is affected many-fold compared with paid productivity [30].

The HAQ functional disability score as the most significant identifier of work disability was confirmed in the QUEST-RA database including different countries and cultures in addition to what has been observed in Western countries [31,32]. In addition to disease activity and damage, variables such as age, sex, education, occupation, duration of disease, functional status, family, physical demand, and time control issues at work have an impact on work status [1,33]. In one study, if scores were known for physical function, occupation, age, and duration of disease, other clinical status measures, including joint counts, radiographs, and laboratory tests, did not add to the explanation of a patient's work or disability status [34].

### Limitations

This study has several limitations. First, cross-sectional QUEST-RA data cannot provide detailed analysis of all work disability-related issues such as specified instruments concerning work disability/participation but rather provide an overview across various countries. The short 4-page patient questionnaire did not include a question about workload, for example, to analyze in this multinational setting whether, as has been suggested [35], work disability is a consequence of mismatch between an individual's functional capacity (HAQ) and work requirements. Second, some of the patients have a long disease duration and might not fully remember their work history. Therefore, recall bias may be one of the limitations of this study. Third, a limited number of patients were included per country. Nonetheless, results were quite similar among clinics within each country [20]. Fourth, it is possible that only RA patients with poor clinical status visit clinics in low-GDP countries and that patients with better status in high-GDP countries seek medical care. While this possibility cannot be excluded, the study was designed to incorporate a cross-section of patients seen in various countries. Nevertheless, the QUEST-RA results illustrate the potential value of collaborative databases, with identical database content and architecture, to provide an opportunity to study associations between disease characteristics, therapies, and outcomes at many sites in many countries with different cultural and socioeconomic environments. Work disability accounts for a major fraction of the costs of RA to both patients and societies. Improved work disability outcomes in RA will require attention to social, economic, and political issues and wider physician and public education in addition to improved medical management of the disease.

## Conclusions

Work disability rates remain high among people with RA during this millennium. In low-GDP countries, people remain working with high levels of disability and disease activity. Cultural and economic differences between societies affect work disability as an outcome measure for RA.

## Abbreviations

ACR20: American College of Rheumatology 20% improvement criteria; DAS28: disease activity score using 28 joint counts; DMARD: disease-modifying antirheumatic drug; ESR: erythrocyte sedimentation rate; FIN-RACo: The Finnish Rheumatoid Arthritis Combination Trial; GDP: gross domestic product; HAQ: Health Assessment Questionnaire; ln: logarithm; PTGL: patient global estimate of disease activity; QUEST-RA: Quantitative Standard Monitoring of Patients with Rheumatoid Arthritis; RA: rheumatoid arthritis; RF: rheumatoid factor; SJC28: swollen joint count using 28 joints; TJC28: tender joint count using 28 joints; USD: US dollars.

## Competing interests

One or more authors of this article have received reimbursements, fees, or funding from the following pharmaceutical companies: Abbott (Abbott Park, IL, USA), Allergan, Inc. (Irvine, CA, USA), Amgen (Thousand Oaks, CA, USA), Bristol-Myers Squibb Company (Princeton, NJ, USA), Chelsea Therapeutics, Inc. (Charlotte, NC, USA), GlaxoSmithKline (Uxbridge, Middlesex, UK), Jazz Pharmaceuticals (Palo Alto, CA, USA), Merrimack Pharmaceuticals, Inc. (Cambridge, MA, USA), MSD (Whitehouse Station, NJ, USA), Pfizer Inc (New York, NY, USA), Pierre Fabre Medicament (Boulogne Cedex, France), Roche (Basel, Switzerland), sanofi-aventis (Paris, France), Schering-Plough Corporation (Kenilworth, NJ, USA), UCB (Brussels, Belgium), and Wyeth (Madison, NJ, USA).

## Authors' contributions

TS, HK, and TP contributed to study design and analyses. The entire QUEST-RA study group contributed to data collection and preparation of the manuscript. All authors read and approved the final manuscript.
